# Angiotensin II and TGFB1 Induce Global DNA Demethylation and Telomere Elongation in Bovine Embryos

**DOI:** 10.3390/ani16162548

**Published:** 2026-08-14

**Authors:** Shunsuke Hara, Kanami Abe, Minori Shida, Komei Shirasuna, Hisataka Iwata

**Affiliations:** 1 Department of Animal Science, Tokyo University of Agriculture, Setagaya City, Tokyo 156-8502, Japan; syunsuke.khantrast@gmail.com (S.H.); euph.hamham01@gmail.com (K.A.); minori130617@outlook.jp (M.S.); ks205312@nodai.ac.jp (K.S.); 2 Department of Livestock Reproduction Research, Institute of Livestock and Grassland Science, National Agriculture and Food Research Organization, Tsukuba 305-0901, Japan

**Keywords:** angiotensin II (Ang II), transforming growth factor-beta 1 (TGFB1), telomere length, growth factor, DNA methylation, bovine embryo

## Abstract

Telomere length (TL) is an important indicator of embryo quality and is associated with subsequent developmental outcomes. However, little is known about the factors that regulate TL during embryonic development. Because in vitro-produced embryos typically exhibit shorter TL than in vivo-developed embryos, this study aimed to identify factors that regulate TL in bovine embryos. Candidate regulators were identified by integrating RNA-seq data from bovine blastocysts with normal or short TL and information on factors present in oviductal fluid. Ingenuity Pathway Analysis of differentially expressed genes identified angiotensin II (Ang II), transforming growth factor-beta 1 (TGFB1), and epidermal growth factor (EGF) as candidate regulators. The effects of supplementing these factors in the culture medium were then evaluated with respect to embryonic development, TL, telomerase activity, and DNA methylation. All three factors improved embryonic development. Ang II and TGFB1 increased TL at the blastocyst stage and reduced global DNA methylation levels at both the 8-cell and blastocyst stages. Transcriptomic analysis further suggested that Ang II and TGFB1 modulated pathways related to chromatin remodeling and telomere maintenance. These findings identify Ang II and TGFB1 as important regulators of telomere length during bovine preimplantation development and suggest that their effects are mediated, at least in part, through epigenetic remodeling.

## Simple Summary

Telomere length (TL) is an important marker of embryo quality, but the factors affecting TL during embryonic development are not well understood. In vitro-produced embryos exhibit shorter TL than in vivo-developed embryos. In this study, we investigated factors that may extend TL in bovine embryos. Using RNA-seq analysis, we identified three candidate factors: angiotensin II (Ang II), transforming growth factor-beta 1 (TGFB1), and epidermal growth factor (EGF). Supplementation with these factors improved embryonic development only when administered from the zygote to the 8-cell stage, whereas treatment initiated after the 8-cell stage produced no detectable phenotypic changes. Early treatment also induced global DNA demethylation and enhanced telomere elongation at the blastocyst stage. Concomitantly, reduced levels of protection of telomeres 1 (POT1) and increased histone acetylation levels were observed in embryos. These findings suggest that Ang II and TGFB1 support embryonic development in association with epigenetic modifications and telomere elongation.

## 1. Introduction

In vitro-produced (IVP) embryos are widely used in human assisted reproductive technologies and livestock production. However, the quality of IVP embryos remains inferior to that of in vivo-developed embryos. Because the efficiency and quality of IVP embryos are critical determinants of reproductive success in cattle, bovine embryos provide an important model for investigating the mechanisms that regulate embryonic development and embryo quality. In particular, the lower quality of in vitro-produced embryos is associated with reduced pregnancy rates and an increased incidence of abnormal fetal and placental development in both humans [1,2] and cows [3,4,5,6]. One approach to improving the quality of IVP embryos is to supplement the culture medium with biologically active molecules derived from the reproductive tract [6]. In this context, cytokines and growth factors present in the oviduct have been reported to enhance embryonic development and improve embryonic quality [7,8,9,10,11].

Telomeres are dynamic structures that cap the ends of mammalian chromosomes and preserve genomic integrity. They consist of tandem repeats of a guanine-rich sequence (5′-TTAGGG-3′) and the shelterin complex, which is composed of six telomere-associated proteins (Protection of telomeres 1 (POT1), Telomere protection protein 1 (TPP1), TERF1-intaracting nuclear factor 2 (TIN2), Telomeric repeat-binding factor 1 (TRF1), Telomeric repeat-binding factor 2 (TRF2), and Repressor/activator protein 1 (RAP1)). These proteins regulate telomerase accessibility and telomere length (TL) homeostasis during embryonic development [12,13]. TL progressively shortens with each cell division due to the end-replication problem. In embryos, TL can be elongated by alternative lengthening of telomeres (ALT) and telomerase [14]. Emerging evidence suggests that TL is closely linked to embryo quality and developmental competence. In human clinical data, TL in embryos is associated with implantation potential [15], whereas telomere shortening is associated with embryonic fragmentation and aneuploidy [14,16]. Also, factors including oxidative stress and intracellular NAD^+^ (Nicotinamide adenine dinucleotide) levels affect TL in embryos cultured in vitro [17,18]. Furthermore, offspring conceived through in vitro fertilization have been reported to exhibit shorter TL compared with those conceived naturally [19]. However, it remains unclear whether physiological factors present in the oviduct regulate TL during early embryonic development.

Epigenetic regulation, including DNA methylation and histone modification, is known to influence chromatin structure and may affect TL. DNA methylation is a fundamental epigenetic mechanism regulating embryonic development, and it is reported that in vitro-developed embryos have higher DNA methylation levels than their in vivo counterparts [20,21,22]. A previous study examined the relationship between embryonic quality and TL in embryos, using a telomerase inhibitor (TMPyP4; Tetrakis(1-methylpyridinium-4-yl)porphyrin) to produce embryos with shortened telomeres. They conducted RNA-seq to examine embryonic quality and deposited the RNA-seq data in a publicly available database [23]. However, these RNA-seq data have not been analyzed to identify candidate factors involved in telomere elongation.

The present study had two objectives. First, we screened physiological oviduct-derived factors that regulate telomere length in bovine embryos by analyzing publicly available RNA-seq data. Second, we investigated the epigenetic mechanisms through which these factors regulate telomere length, focusing on DNA methylation and telomere-associated pathways.

## 2. Materials and Methods

### 2.1. Collection of Bovine Ovaries and Oocytes

Bovine ovaries were collected from cows (hybrid of Japanese Black bull and Holstein cows, aged 28 months, 700–800 kg) at a slaughterhouse. The ovaries were transported to the laboratory within 4 h in phosphate-buffered saline (PBS) containing 100 IU/mL penicillin and 0.1 µg/mL streptomycin at 25 °C. Cumulus cell-oocyte complexes (COCs) were collected from antral follicles (3–5 mm in diameter) using an 18 G needle (Terumo, Tokyo, Japan) connected to a 10 mL syringe (Terumo). This study was approved by the Ethics Committee for Animal Experiments of Tokyo University of Agriculture (approval number: 250065).

### 2.2. Chemicals and Media

All the chemicals were purchased from Nacalai Tesque (Kyoto, Japan) unless otherwise stated. In vitro maturation (IVM) medium was TCM199 (Gibco, Grand Island, NY, USA) supplemented with 10 mM taurine, 10 ng/mL EGF (Sigma-Aldrich, St. Louis, MO, USA), 100 IU/mL penicillin, 0.1 µg/mL streptomycin, and 10% fetal calf serum (FCS; 5703H; ICN Pharmaceuticals, Costa, Mesa, CA, USA). In vitro fertilization (IVF) medium was synthetic oviductal fluid (SOF) supplemented with 3 mg/mL fatty acid-free bovine serum albumin (BSA) and 10 IU/mL heparin [24]. In vitro culture (IVC) medium was SOF supplemented with essential and non-essential amino acids (Sigma-Aldrich).

### 2.3. In Vitro Maturation and In Vitro Fertilization

COCs were cultured with IVM medium for 21 h. COCs were then fertilized with frozen–thawed semen of a Japanese Black bull. The semen was washed with the discontinuous gradient Percoll solution (Cytiva, Tokyo, Japan, 30, and 60% in IVF medium) and co-cultured with COCs at a concentration of 1 × 10^6^ sperm/mL for 6 h. Then, COCs were cultured in IVC medium for 12 h post-insemination.

### 2.4. Prediction of Candidates Associated with Telomere Elongation and Treatment of Zygotes

Differentially expressed genes (DEGs) were identified using publicly available RNA-seq data of the bovine blastocyst-stage embryos (BioProject accession number: PRJDB17567), in which bovine zygotes were cultured in the presence of 10 nM TMPyP4 (telomerase inhibitor) or vehicle (water) until the blastocysts used the same protocol as that used in the present study. TMPyP4 treatment resulted in shortened TL in embryos. Raw sequence data were downloaded and processed by trimming, reference genome mapping, and differentially expressed genes were identified using CLC Genomics Workbench version 23.0.2 (Qiagen, Hilden, Germany). A total of 614 DEGs (list of all DEGs in Table S1: TMPyP4-DEGs, absolute fold change > 2, *p* < 0.05) were found and subjected to upstream regulator analysis using Ingenuity Pathway Analysis (IPA; Qiagen) to identify inhibited upstream regulators (list of all upstream regulators in Table S2: TMPyP4-IPA, predicted to be inhibited with TMPyP4 treatment). Growth factors were selected from the top-ranked upstream regulators based on their reported presence in oviductal fluid [25,26,27]. Since TMPyP4 induces telomere shortening, upstream regulators predicted by IPA to be inhibited in TMPyP4-treated embryos were regarded as candidate physiological factors that positively regulate telomere maintenance or elongation.

### 2.5. Treatment of Embryos with Candidate Growth Factors

Embryos were cultured in IVC medium supplemented with 3 mg/mL fatty acid-free BSA containing either vehicle (control; 1:1000 dilution of water), 1.0 pg/mL Ang II (Sigma-Aldrich), 10 ng/mL TGFB1 (FUJIFILM Wako Pure Chemical Corporation, Osaka, Japan), or 10 ng/mL EGF (Sigma-Aldrich). The concentrations of these factors were determined based on previous reports, as described in the Experimental Design section (Section 2.10.2). The total cell number of blastocyst-stage embryos was determined following Hoechst 33342 staining using a fluorescence microscope (Keyence, Tokyo, Japan).

### 2.6. Quantitative PCR Analysis of Telomere Lengths (TL)

TL was measured in duplicate using real-time polymerase chain reaction (PCR), as previously described [24]. Randomly selected individual 8-cell and blastocyst-stage embryos were analyzed. Real-time PCR was performed using KAPA SYBR FAST qPCR Master Mix (Kapa Biosystems, Wilmington, MA, USA), 0.5 μM primer sets (5′-CGGTTTGTTTGGGTTTGGGTTTGGGTTTGGGTTTGGGTT-3′ and 5′-GGCTTGCCTACCCTTACCCTTACCCTTACCCTTACCCT-3′), and a CFX Connect Real-Time PCR Detection System (Bio-Rad Laboratories, Hercules, CA, USA). The PCR conditions were as follows: 95 °C for 3 min, followed by 39 cycles of 98 °C for 5 s and 59 °C for 60 s. Standard curves were generated using serial dilutions representing copies of the external standards (a synthesized oligonucleotide; 90 bp in length; TTAGGG, repeated 15-times). TL was represented as relative TL, where the TL in the control group was set at 1.0. All PCR efficiencies were greater than 1.99–2.0. Measurement of TL in the 8-cell stage or blastocyst stage was conducted using one 96-well plate to avoid inter-plate variation in measurements. Therefore, results were presented as one set (control vs. additional group) for each stage and factor.

### 2.7. Immunostaining

Embryos were fixed in 4% paraformaldehyde in PBS for 20 min, and then permeabilized in 0.2% Triton X-100 for 30 min. The embryos were blocked in PBS containing 5% BSA for 1 h. Subsequently, these embryos were incubated with a primary anti-rabbit polyclonal anti-5-methylcytosine antibody (5mC: D3S2Z, Cell Signaling, Danvers, MA, USA; Cat# 28692S), rabbit polyclonal anti-acetyl-lysine (abcam, Cambridge, UK; Cat# ab80178) and rabbit polyclonal anti-protection of telomeres 1 (POT1: Proteintech, Rosemont, IL, USA; Cat# 10581-1-AP) overnight, followed by treatment with a secondary Alexa-Fluor-555-conjugated anti-rabbit IgG Fab2 antibody (Cell Signaling; Cat# 4413S). For 5mC staining, embryos were treated with 2 N HCl for 1 h before blocking. The embryos were mounted on a slide and observed using a fluorescence microscope (Leica DMI6000 B fluorescence microscope equipped with LAS AF software, Leica, Wetzlar, Germany). The fluorescence intensity of the nuclear region was quantified as 5mC, acetyl-lysine and POT1. Fluorescence intensities were quantified using ImageJ software ver. 1.54g (NIH, Bethesda, MD, USA).

### 2.8. Transcriptomic Analysis of Embryos Treated with Ang II and TGFB1

DEGs were identified from publicly available RNA-seq data of the bovine blastocyst-stage embryos (BioProject accession number: PRJDB39766), where bovine zygotes were cultured in the absence or presence of 1.0 pg/mL Ang II or 10 ng/mL TGFB1 until the 8-cell stage, after which embryos were cultured in the absence of these factors until the blastocyst stage. This protocol was identical to that used in the present study. The raw data were downloaded and subjected to trimming, reference genome mapping, and DEG identification using CLC Genomics Workbench ver. 23.0.2 (Qiagen, Hilden, Germany). In addition, 2 DEG datasets (between control vs. Ang II and between control vs. TGFB1) were compared to determine overlapping genes. These genes were subjected to pathway analysis (Kyoto Encyclopedia of Genes and Genomes; KEGG) and upstream regulator analysis (IPA, Qiagen). KEGG pathway enrichment analysis was performed using the Database for Annotation, Visualization and Integrated Discovery (DAVID; https://david.ncifcrf.gov, accessed on 15 January 2026), with *Bos taurus* used as the background species. The data (raw FASTQ file format RNA-seq data) presented in this study are openly available from the DNA Data Bank of Japan with links to the PRJDB17567 (the blastocyst with or without TMPyP4) and PRJDB39766 (the blastocyst between control vs. Ang II and control vs. TGFB1) in the DNA Data Bank of Japan (DDBJ) BioProject database.

### 2.9. Measurement of Telomerase Activity in Blastocysts

Blastocyst-stage embryos derived from Ang II- or TGFB1-treated embryos were examined for telomerase activity. Telomerase activity was measured using the TRAPeze Telomerase Detection Kit (Merck, Darmstadt, Germany) according to the manufacturer’s protocol. Fifteen embryos were used for each replicate, and a total of 3 samples were prepared for each group. The band images were observed and captured using ImageQuant LAS 4000 ver. 1.2 (GE Healthcare, Chicago, IL, USA). The fluorescence intensities were quantified using ImageJ software (NIH, Bethesda, MD, USA).

### 2.10. Experimental Design

#### 2.10.1. Identification of Candidate Factors Potentially Promoting TL Elongation in Embryos

Candidate factors potentially involved in the maintenance or elongation of embryonic TL were identified based on previously published RNA-seq data, IPA, and information on oviductal fluid components.

#### 2.10.2. Effect of Early Treatment with Ang II and TGFB1 on Global DNA Demethylation and Telomere Elongation

The effects of treatment with candidate growth factors (Ang II, TGFB1, and EGF) from the zygote to the 8-cell stage (hereafter referred to as early treatment) on embryonic development, DNA methylation, and TL were examined. Based on previous studies, the concentration of these factors was determined (Ang II; [26], TGFB1; [28], and EGF; [8]). Approximately 20 embryos were used per replicate, and the experiment was repeated 7 times. For each analysis, approximately 22 randomly selected 8-cell-stage embryos were used for DNA methylation and TL assessment.

#### 2.10.3. Effect of Late Treatment with Ang II and TGFB1 on Global DNA Demethylation and TL Elongation

The treatment period was shifted from the zygote-to-8-cell stage (early treatment) to the 8-cell-to-blastocyst stage (late treatment), and the effects of Ang II and TGFB1 were evaluated as described in Section 2.10.2.

#### 2.10.4. Effect of Ang II- or TGFB1 on Protein Acetylation, POT1 Expression, and Expression of Genes Regarding Telomere

To identify molecules potentially mediating the effects of Ang II and TGFB1, we performed IPA and pathway analyses using publicly available transcriptomic data from the DDBJ BioProject (accession number: PRJDB39766). The dataset consisted of blastocysts derived from embryos treated with Ang II or TGFB1 from zygote to the 8-cell stage. Embryos were produced using the same protocol as that employed in the present study.

#### 2.10.5. Effect of Ang II and TGFB1 Treatment on Protein Acetylation, POT1 Expression and Telomerase Activity

Based on the RNA-seq data analysis (Experiment 4), we first examined the effects of early treatment with Ang II and TGFB1 (from the zygote to the 8-cell stage) on the acetylated lysine levels in 8-cell-stage embryos and on POT1 expression in blastocysts. We then investigated the effects of Ang II and TGFB1 treatment from the zygote to the 8-cell stage on telomerase activity in blastocysts.

### 2.11. Statistical Analysis

Data normality was assessed using the Shapiro–Wilk test. Parametric or nonparametric tests were then selected accordingly. Parametric data were analyzed using the Student’s t-test and nonparametric data were analyzed using the Mann–Whitney U test. The cleavage and blastocyst formation rates were arcsine-transformed prior to analysis. All data are presented as mean ± standard error of the mean (SEM). Statistical significance was set at *p* < 0.05.

## 3. Results

### 3.1. Early Treatment of Ang II and TGFB1 Induces Global DNA Demethylation and Elongate TL

We predicted factors affecting TL in embryos (Table 1) based on the presence in the oviductal fluid and IPA of DEGs comparing embryos with long and short telomeres. Of these factors, we selected angiotensin II (Ang II), a downstream effector of AGT, transforming growth factor beta 1 (TGFB1), and epidermal growth factor (EGF).

### 3.2. Ang II and TGFB1 Treatment from the Zygote to the 8-Cell Stage Induces Global DNA Demethylation and Promotes Telomere Elongation

**Figure 1:**
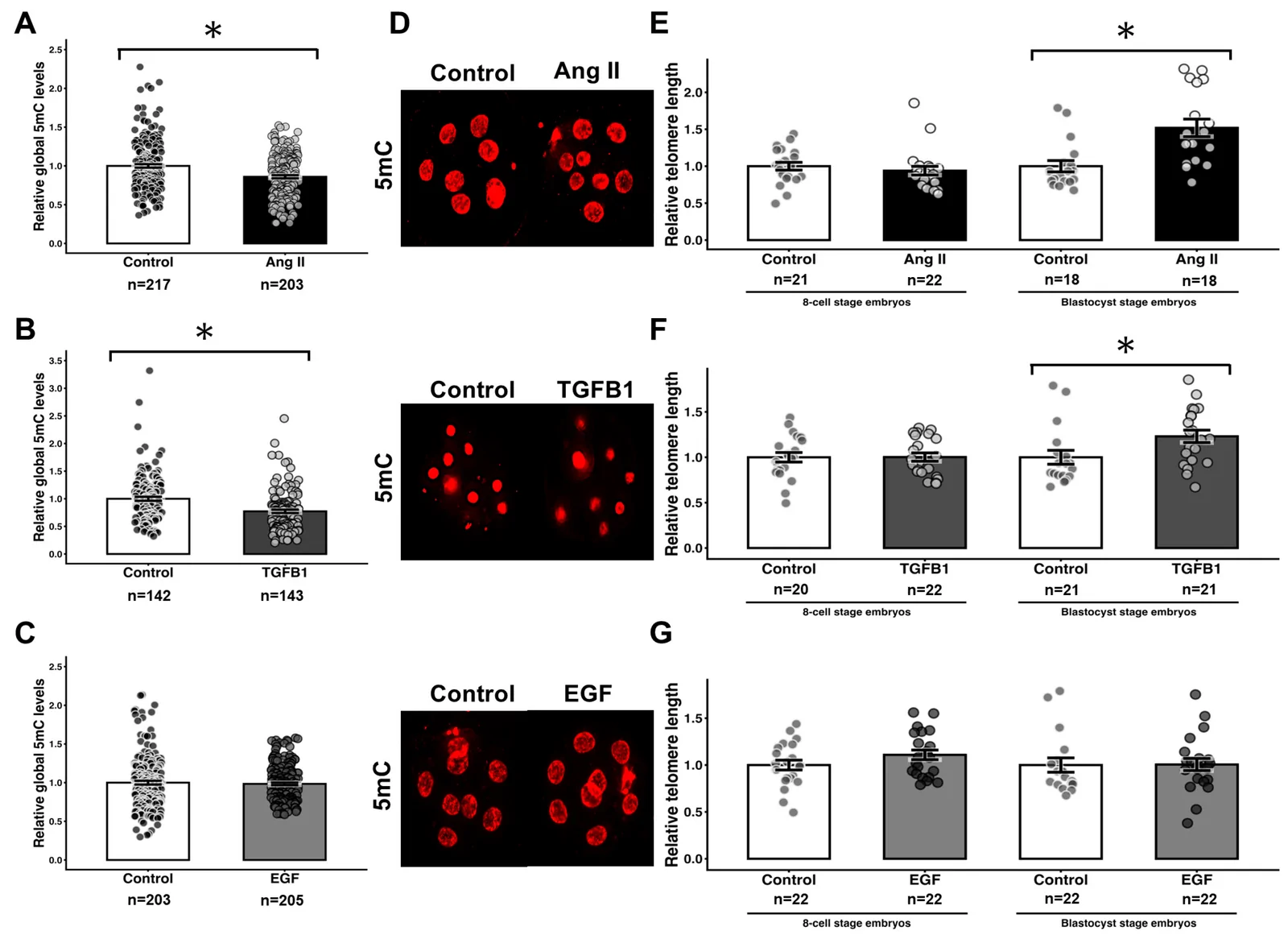
Effects of early treatment of candidate growth factors on DNA methylation and TL: Relative global 5mC levels in the 8-cell stage embryos developed with vehicle, Ang II (**A**), TGFB1 (**B**) or EGF (**C**), and representative images of embryos (**D**). Relative TL at the 8-cell and blastocyst-stage embryos treated with Ang II (**E**), TGFB1 (**F**) or EGF (**G**). (*n* =); number of embryos examined. The expression level of the control was set at 1.0. * *p* < 0.05.

Supplementation of the culture medium with each candidate growth factor (Ang II, TGFB1, or EGF) improved embryonic development, as reflected by an increased blastocyst formation rate and/or a higher total cell number in blastocysts (Table 2). Treatment with Ang II or TGFB1 significantly reduced global DNA methylation (5mC) levels at the 8-cell stage (Figure 1A,B), whereas EGF treatment had no effect on DNA methylation (Figure 1C). Reduced DNA methylation levels were also observed at the blastocyst stage following Ang II or TGFB1 treatment (Supplementary Figure S1A,B). Embryos treated with either Ang II or TGFB1 exhibited longer telomeres at the blastocyst stage, whereas no difference in TL was detected at the 8-cell stage (Figure 1E,F). In contrast, EGF treatment did not affect TL at either the 8-cell or blastocyst stage (Figure 1G).

### 3.3. Ang II and TGFB1 Treatment from the 8-Cell to Blastocyst Stage Does Not Induce Global DNA Demethylation or Telomere Elongation

**Figure 2:**
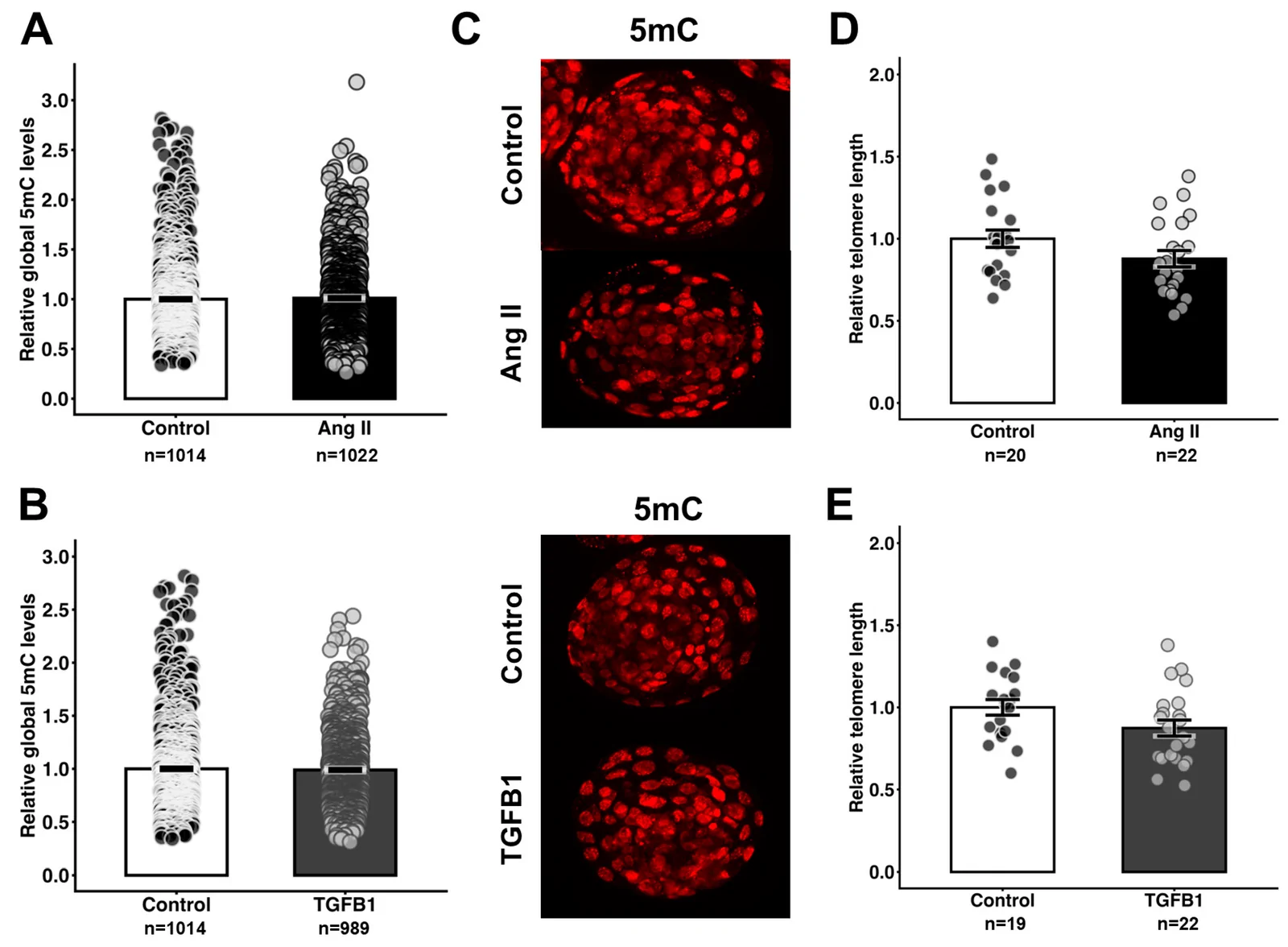
Effects of Ang II and TGFB1 treatment from the 8-cell stage to the blastocyst stage on DNA methylation and telomere length in blastocysts. Relative global 5mC levels in blastocyst-stage embryos treated with vehicle, Ang II (**A**), or TGFB1 (**B**) from the 8-cell stage onward, and representative images of blastocysts (**C**). Relative telomere length in blastocyst-stage embryos treated with vehicle, Ang II (**D**), or TGFB1 (**E**). (*n* =); number of embryos analyzed. The value of the vehicle control group was set to 1.0.

Ang II treatment from the 8-cell stage to the blastocyst stage increased the total cell number of blastocysts. Similarly, TGFB1 treatment increased the blastocyst formation rate (Table 3). However, neither Ang II nor TGFB1 affected global DNA methylation levels (Figure 2A,B) or TL in blastocyst-stage embryos compared with the untreated control group (Figure 2D,E).

### 3.4. Transcriptomic Analysis of Ang II- or TGFB1-Treated Embryos

**Figure 3:**
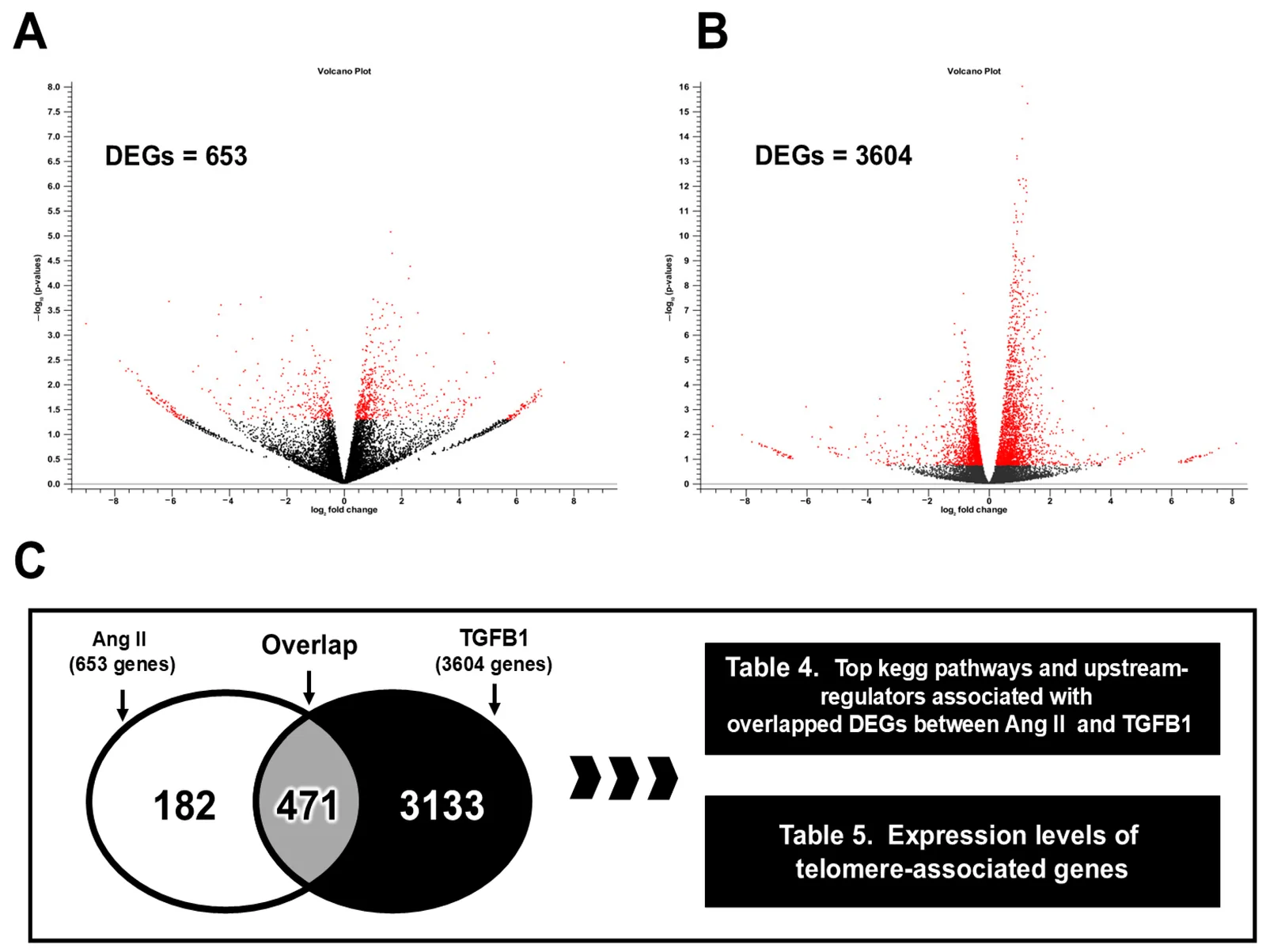
Transcriptomic analysis of Ang II- or TGFB1-treated embryos: Volcano plot of differentially expressed genes (DEGs) of Ang II versus control (**A**) and of TGFB1 versus control (**B**). Red dots indicate significant differences (*p* < 0.05, TPM > 0.1), whereas black dots indicate no significant differences. Venn diagram showing overlapping DEGs between Ang II vs. control and TGFB1 vs. control (**C**).

RNA-seq analysis identified 653 DEGs (Table S3: Ang II-DEGs) between Ang II and control groups, and 3604 DEGs (Table S4: TGFB1-DEGs) between TGFB1 and control groups (*p* < 0.05, TPM > 0.1; Figure 3A,B). As shown in Figure 3C, 471 genes (Table S5: overlapped DEGs list) overlapped between the 2 DEG groups. The overlapped DEGs were associated with the sphingolipid signaling pathway, mitophagy, and autophagy (list of all overlapped pathways in Table S6: overlapped pathway and upstream regulator of interest, Table 4). In addition, MITF, FIRRE, and Trichostatin A (TSA; a histone deacetylase inhibitor) were the top activated upstream regulators (Table 4; list of all upstream regulators in Table S7: overlapped IPA). Focusing on telomere-associated genes, Ang II or TGFB1 treatment reduced expression levels of POT1 (Table 5).

### 3.5. Ang II and TGFB1 Treatment Induce Histone Acetylation and Reduce POT1 Expression

**Figure 4:**
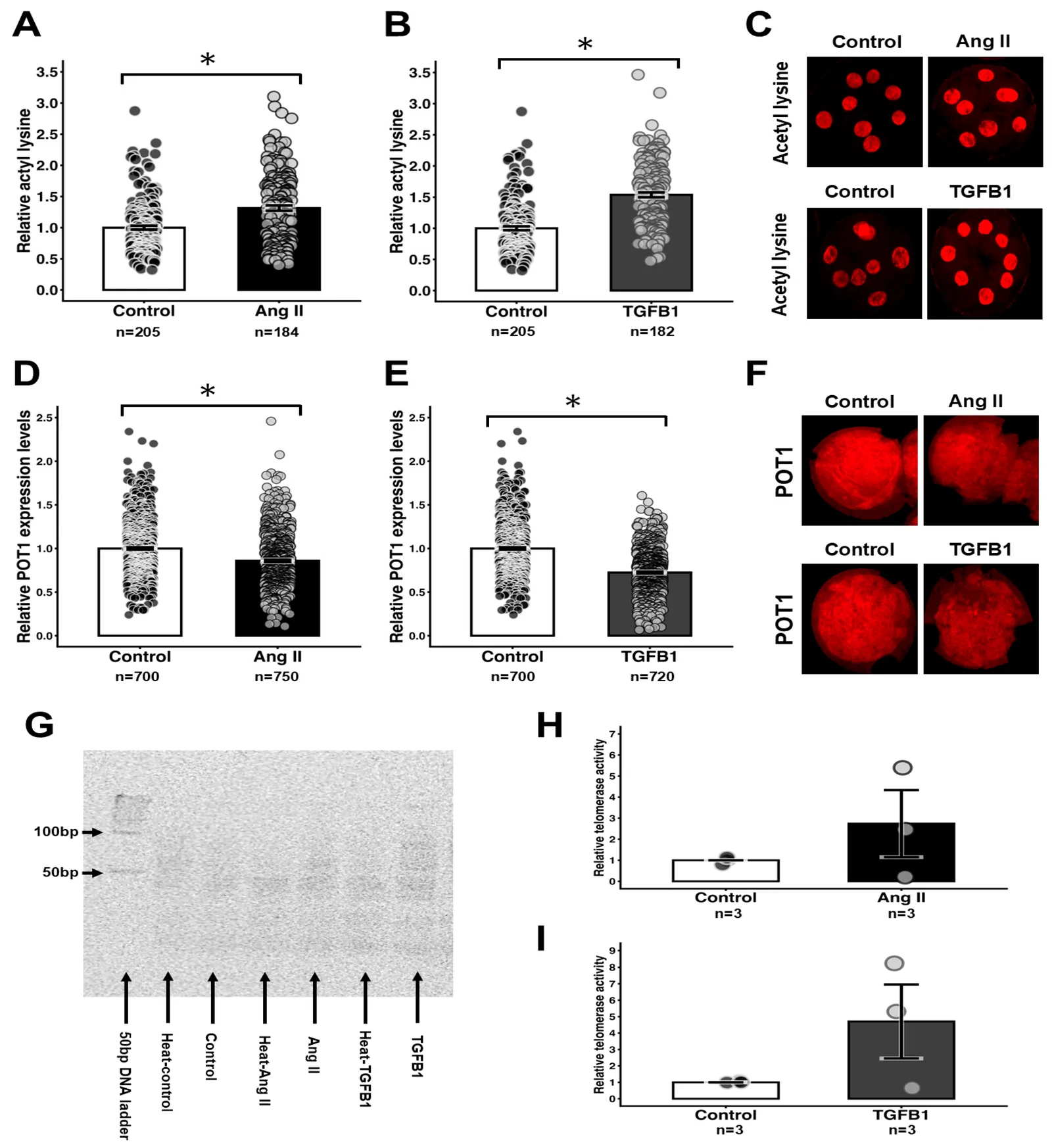
Effects of Ang II and TGFB1 on acetylated lysine levels and POT1 expression. Relative acetylated lysine levels in 8-cell-stage embryos treated with Ang II (**A**) or TGFB1 (**B**), and representative images of the embryos (**C**). Relative POT1 protein levels in blastocyst-stage embryos derived from embryos treated with Ang II (**D**) or TGFB1 (**E**) from zygote to the 8-cell stage, and representative images of the embryos (**F**). Representative PAGE gel images of telomerase activity (**G**). Relative telomerase activity in the blastocyst-stage embryos treated with Ang II (**H**) or TGFB1 (**I**). (*n* =); The number of blastomeres examined. Heat-sample: negative telomerase sample. The control was set at 1.0. * *p* < 0.05.

Ang II and TGFB1 treatment increased histone acetylation levels at the 8-cell stage, supporting the prediction that TSA is the top upstream regulator (Figure 4A,B). Notably, both treatments significantly reduced POT1 expression at the blastocyst stage, consistent with RNA-seq findings (Figure 4D,E). However, telomerase activity (Table S8: Trap assay) at the blastocyst stage did not differ between Ang II- or TGFB1-treated embryos and control (Figure 4H,I).

## 4. Discussion

The present study demonstrated that treatment with Ang II and TGFB1 before zygotic genome activation significantly altered embryonic development, including global DNA demethylation and telomere elongation.

IPA is a knowledge-based analytical approach that predicts upstream regulators that may explain the observed gene expression changes. In the present study, Ang II, TGFB1, and EGF were predicted to be activated upstream regulators that may promote telomere maintenance or elongation. These molecules have been reported to be present in oviductal fluid and to promote embryonic development [26,27]. Consistent with these reports, our results demonstrate that these factors improved embryonic development.

Notably, our study provides the first in vitro evidence in bovine embryos that Ang II and TGFB1 exert an additional effect on telomere elongation during preimplantation development. Increased TL was observed at the blastocyst stage but not at the 8-cell stage, suggesting that telomere elongation occurs during the later stages of development (from the 8-cell stage to the blastocyst stage). Interestingly, treatment before the 8-cell stage resulted in TL elongation, whereas treatment after the 8-cell stage did not. Considering that TL elongation after zygotic genome activation (8-cell stage in cows) largely depends on telomerase-mediated extension [29,30], these findings suggest that Ang II and TGFB1 exposure during early development may prime embryos for subsequent telomere elongation around the blastocyst stage.

Previous studies have suggested an inverse relationship between TL elongation and DNA methylation during embryonic development [31,32]. Global DNA demethylation is a critical process during early cleavage-stage development and forms the foundation for subsequent embryonic development and differentiation [33]. In line with this notion, we found that treatment of early-stage embryos with Ang II and TGFB1 accelerated global DNA demethylation, and this lower methylation status persisted until the blastocyst stage. Previous studies have reported that in vitro-produced embryos exhibit altered epigenetic profiles, including higher DNA methylation levels and shorter telomeres compared with their in vivo-derived counterparts [20,21]. Furthermore, accumulating evidence indicates that in vitro culture conditions, including culture media composition and its components, can influence DNA methylation patterns in embryos and fetuses [34,35]. Together with these reports, our findings suggest that Ang II and TGFB1 may contribute to modulating the epigenetic status of in vitro-produced embryos and may represent potential candidates for reducing epigenetic differences between in vitro- and in vivo-developed embryos.

In the present study, no significant differences in telomerase activity were detected between factor-treated and control embryos. Furthermore, RNA-seq analysis revealed that Ang II and TGFB1 treatment did not alter the mRNA expression levels of telomerase reverse transcriptase (TERT). These results suggest that TL elongation may not be driven by increased telomerase expression or activity. Therefore, the observed telomere elongation is more likely to result from altered accessibility of telomeres to the telomerase complex than from increased telomerase abundance. Supporting this idea, we observed reduced expression of POT1 in treated embryos. Consistently, it is reported that POT1 is known to inhibit telomerase access to telomeres [36,37].

Additionally, comparison of the DEGs between control and Ang II-treated embryos and between control and TGFB1-treated embryos revealed a set of overlapping genes. First, shared DEGs were enriched in sphingolipid signaling and mitophagy. Previous studies have shown that nuclear sphingosine-1-phosphate regulates chromatin remodeling through inhibition of histone deacetylase 1/2 (HDAC1/2), whereas mitochondrial homeostasis influences epigenetic regulation by altering cellular metabolic intermediates required for chromatin-modifying enzymes [38]. Therefore, these pathways may indirectly contribute to telomere-associated epigenetic regulation, although the precise molecular mechanisms remain to be clarified. Next, IPA of these shared DEGs identified TSA as the top predicted activated upstream regulator. Interestingly, TSA treatment has previously been reported to induce telomere elongation in embryos [39]. TSA is a histone deacetylase inhibitor that promotes histone acetylation and transcriptionally active chromatin. Consistently, our experiments confirmed that Ang II and TGFB1 treatment increased histone acetylation levels in embryos. Furthermore, IPA revealed that FIRRE is a top upstream regulator. FIRRE has been reported to be a key factor regulating chromatin accessibility [40]. Taken together, our results suggest that Ang II and TGFB1 are important factors that not only enhance embryonic development but also modulate epigenetic states, including DNA methylation and histone acetylation. These epigenetic changes may be associated with telomere maintenance by facilitating the interaction between telomerase and telomeric chromatin. However, direct evidence that TGFB1- or Ang II-induced chromatin remodeling enhances telomerase accessibility to telomeres is still lacking. Further studies are required to directly examine the relationship between TGFB1- or Ang II-induced chromatin changes and telomerase accessibility. An important remaining question is whether embryos with longer telomeres ultimately result in offspring with increased TL. Because TL is considered a key biomarker associated with lifespan and aging, this possibility warrants investigation in further studies.

Finally, this study has several limitations. First, telomere length was measured only by relative quantitative PCR (qPCR). Although qPCR is widely used in large epidemiological studies, future studies should validate these findings using gold-standard methods, such as terminal restriction fragment (TRF) Southern blot analysis or fluorescence in situ hybridization (FISH), to enable absolute telomere length quantification. Second, the assessment of chromatin and telomere status should be expanded by incorporating more advanced approaches, such as measurements of HDAC activity and chromatin accessibility profiling using Assay for Transposase-Accessible Chromatin using sequencing (ATAC-seq), to strengthen our understanding of the TGFB1- and Ang II-induced changes in chromatin remodeling and telomere elongation. Finally, we examined only a limited range of TGFB1 and Ang II concentrations. Therefore, the optimal and potentially detrimental concentrations as well as combinations of other growth factors for embryonic development and telomere maintenance remain to be determined.

## 5. Conclusions

In conclusion, the present study demonstrates that Ang II and TGFB1 are important modulators associated with enhanced embryonic development and telomere elongation during preimplantation development. Telomere elongation was observed specifically at the blastocyst stage and required exposure to these factors before the 8-cell stage, indicating that the early embryonic environment is critical for subsequent telomere dynamics. Importantly, telomere elongation was not associated with increased telomerase activity or TERT expression but was accompanied by epigenetic changes that may be associated with a chromatin state favorable for telomere elongation. Specifically, Ang II and TGFB1 treatment induced global DNA demethylation, reduced POT1 expression, and increased histone acetylation, collectively suggesting the establishment of a chromatin environment that is more permissive for telomere elongation. These findings provide new insights into the mechanisms by which the early embryonic environment regulates telomere biology through epigenetic remodeling. Furthermore, they highlight the potential importance of oviduct-derived factors in improving not only embryonic developmental competence but also the long-term biological quality of embryos. Together, these findings support a model in which oviduct-derived factors promote telomere elongation by modulating the epigenetic landscape of preimplantation embryos rather than by increasing telomerase expression or activity. Future studies are needed to determine whether the telomere alterations induced by Ang II and TGFB1 persist during later developmental stages and to elucidate their long-term biological significance.

## Figures and Tables

**Table 1:** Top 10 upstream regulators of growth factors enriched by differentially expressed genes in the blastocyst stage embryos produced with or without telomerase inhibitor.

Upstream Regulator	Molecule Type	Activation Score	*p*-Value
TGFB3	growth factor	−3.01	8.53 × 10^−9^
AGT	growth factor	−3.72	1.49 × 10^−8^
TGFB1	growth factor	−3.43	7.11 × 10^−8^
EGF	growth factor	−3.12	1.24 × 10^−6^
TGFB2	growth factor	−3.17	3.83 × 10^−6^
GDF9	growth factor	−2.20	3.50 × 10^−4^
VEGFA	growth factor	−2.10	6.33 × 10^−4^
LEP	growth factor	−2.79	8.65 × 10^−4^
KITLG	growth factor	−2.14	3.59 × 10^−3^

Activation score indicates the predicted similarity in gene expression changes to those induced by telomerase inhibitor treatment.

**Table 2:** The effect of treatment of embryos with candidate growth factors from the zygote to the 8-cell stage on embryonic development.

Group	No. of		Cleaved Rate	Blastocyst Stage Embryos	
	Trials	Embryos	(%)	Dev. Rate (%)	Total Cell Number
Control	7	140	53.6 ± 0.9a (*n* = 7)	30.7 ± 1.7a (*n* = 7)	103.1 ± 6.1 (*n* = 32)
Ang II	7	142	56.9 ± 1.1b (*n* = 7)	35.1 ± 0.9b (*n* = 7)	102.6 ± 4.6 (*n* = 32)
Control	8	162	59.2 ± 4.5a (*n* = 7)	33.8 ± 4.0 (*n* = 7)	93.0 ± 4.8a (*n* = 35)
TGFB1	8	159	73.6 ± 4.1b (*n* = 7)	40.7 ± 3.1 (*n* = 7)	119.0 ± 5.5b (*n* = 38)
Control	7	136	64.0 ± 1.2 (*n* = 7)	27.7 ± 2.5a (*n* = 7)	70.0 ± 5.0 (*n* = 38)
EGF	7	136	67.0 ± 1.9 (*n* = 7)	33.9 ± 1.2b (*n* = 7)	71.2 ± 4.5 (*n* = 45)

Embryos were cultured in the medium containing Ang II, TGFB1, EGF, or vehicle for 48 h, and the cleaved rate (≥8-cell) and the developmental (Dev.) rate of the blastocysts were examined on day 2 and day 7 post insemination. Total cell number of the blastocysts was determined. Data were represented as means ± SEM. a–b, *p* < 0.05.

**Table 3:** The effect of Ang II or TGFB1 treatment from the 8-cell to the blastocyst stage on embryonic development.

Group	No. of		Blastocyst Stage Embryos	
	Trials	Embryos	(Dev. Rate %)	(Total Cell Number)
Control	7	87	27.5 ± 1.2 (*n* = 7)	73.7 ± 6.2a (*n* = 24)
Ang II	7	87	29.9 ± 1.3 (*n* = 7)	99.6 ± 6.4b (*n* = 32)
Control	7	87	27.5 ± 1.2a (*n* = 7)	73.7 ± 6.2 (*n* = 24)
TGFB1	7	94	34.0 ± 1.2b (*n* = 7)	79.5 ± 6.3 (*n* = 26)

Embryos were cultured in the medium containing Ang II, TGFB1, or vehicle for 5 days, and the developmental (Dev.) rate of the blastocysts was examined on day 7 post insemination. Total cell number of the blastocysts was determined. Data were represented as means ± SEM. a–b, *p* < 0.05.

**Table 4:** Top KEGG pathways and upstream regulators associated with overlapped DEGs between Ang II and TGFB1.

Name		
**Pathways**		***p*-Value**
Sphingolipid signaling pathway		3.940 × 10^−4^
Mitophagy—animal		6.510 × 10^−4^
Acute myeloid leukemia		7.280 × 10^−4^
Human cytomegalovirus infection		1.230 × 10^−3^
Pancreatic cancer		1.400 × 10^−3^
Autophagy—animal		1.410 × 10^−3^
**Upstream Regulators**	**Status**	***p*-Value**
Microphthalmia-associated transcription factor (MITF)	Activated	1.120 × 10^−9^
Estrogen receptor 1 (ESR1)	Inhibited	1.280 × 10^−8^
Protein tyrosine phosphatase receptor type R (PTPRR)	Inhibited	1.400 × 10^−8^
Firre intergenic repeating RNA element (FIRRE)	Activated	3.880 × 10^−8^
Trichostatin A (TSA)	Activated	4.120 × 10^−7^

Activated: activated upstream regulator causing DEGs in the same direction. Inhibited: Inhibited upstream regulator causing DEGs in the opposite direction.

**Table 5:** The expression levels of telomere-associated genes in Ang II- or TGFB1-treated embryos.

Gene	Ang II/Control		TGFB1/Control	
	Fold Change	*p*-Value	Fold Change	*p*-Value
TERT	−5.28	1.138 × 10^−1^	−3.60	2.293 × 10^−1^
POT1	−1.64	2.000 × 10^−2^	−1.60	7.500 × 10^−3^
TPP1	1.09	5.713 × 10^−1^	1.08	5.082 × 10^−1^
TINF2	−1.02	9.417 × 10^−1^	1.10	5.973 × 10^−1^
TERF1	1.07	6.363 × 10^−1^	1.24	5.704 × 10^−2^
TERF2	−1.06	8.410 × 10^−1^	−1.22	4.458 × 10^−1^
TERF2IP	1.13	5.021 × 10^−1^	1.58	1.049 × 10^−5^

## Data Availability

The data presented in this study are available at https://x.gd/90qIR (accessed on 12 August 2026).

## Supplementary Materials

The following supporting information can be downloaded at: https://www.mdpi.com/article/10.3390/ani16162548/s1, Table S1: TMPyP4-DEG; Table S2: TMPyP4-IPA; Table S3: Ang II-DEGs; Table S4: TGFB1-DEGs; Table S5: overlapped DEGs; Table S6: overlapped pathway; Table S7: overlapped IPA; Table S8: Trap assay; Figure S1: The effect of Ang II and TGFB1 treatment from the zygote to the 8-cell stage embryo on DNA methylation in blastocyst-stage embryos.

## Abbreviations

ALT	alternative lengthening of telomeres
Ang II	Angiotensin II
ATAC-seq	Assay for Transposase-Accessible Chromatin using sequencing
BSA	Bovine serum albumin
COCs	Cumulus cell-oocyte complexes
DAVID	Database for Annotation, Visualization, and Integrated Discovery
DEGs	Differentially expressed genes
DDBJ	DNA Data Bank of Japan
EGF	Epidermal growth factor
ESR1	Estrogen receptor 1
FIRRA	Firre intergenic repeating RNA element
FISH	fluorescence in situ hybridization
HDAC1/2	histone deacetylase 1/2
IPA	Ingenuity pathway analysis
IVC	In vitro culture
IVF	In vitro fertilization
IVM	In vitro maturation
IVP	In vitro production
KEGG	Kyoto Encyclopedia of Genes and Genomes
MITF	Microphthalmia-associated transcription factor
NAD^+^	Nicotinamide adenine dinucleotide
PAGE	Polyacrylamide gel electrophoresis
PBS	phosphate-buffered saline
POT1	Protection of telomeres 1
PTPRR	Protein tyrosine phosphatase receptor type R
qPCR	quantitative PCR
RAP1	Repressor/activator protein 1
RNA-seq	RNA sequencing
SEM	Standard error of the mean
SOF	Synthetic oviductal fluid
TERT	Telomerase reverse transcriptase
TGFB1	Transforming growth factor-beta 1
TIN2	TERF1-interacting nuclear factor 2
TL	Telomere length
TMPyP4	Tetrakis(1-methylpyridinium-4-yl)porphyrin
TPM	Transcripts per kilobase million
TPP1	Telomere protection protein 1
TRF	terminal restriction fragment
TRF1	Telomeric repeat-binding factor 1
TRF2	Telomeric repeat-binding factor 2
TSA	Trichostatin A
5mC	5-methylcytosine
